# Ten-year trends in HIV prevalence among visitors to public health centers under the National HIV Surveillance System in Korea, 2000 to 2009

**DOI:** 10.1186/1471-2458-12-831

**Published:** 2012-09-28

**Authors:** Mee-Kyung Kee, Jin-Hee Lee, Jiyoung Whang, Sung Soon Kim

**Affiliations:** 1 Division of AIDS, Center for Immunology and Pathology, Korea National Institute of Health, Osong, Cheongwon-gun Chungcheongbuk-do, 363-951, South Korea; 2 Division of HIV and TB control, Korea Centers for Disease Control and Prevention, Osong, Cheongwon-gun Chungcheongbuk-do, 363-951, South Korea

**Keywords:** HIV prevalence, HIV surveillance, Voluntary test, Anonymous test, STI risk group

## Abstract

**Background:**

Korea saw a sharp increase in HIV diagnosis from 2000. This serious public health concern must be monitored diligently. We identified the characteristics and trends in HIV prevalence among visitors to public health centers (PHCs) from 2000 to 2009.

**Methods:**

We retrieved ten-year data of HIV tests from 253 PHCs. The HIV prevalence was analyzed by gender, age, nationality, region, and reason for HIV testing. Data were analyzed using logistic regression and score test for trend.

**Results:**

HIV prevalence among PHCs’ visitors has rapidly increased for six years since 2000, decreased from 2006, and then remained stable. Approximately 50% of total HIV tests were performed for sexually transmitted infection risk group (STI RG), who were tested 1.4 times within a year. Women and the 20s comprised approximately 70% and 40% of PHCs’ visitors, respectively. The prevalence of voluntary test takers was the highest and showed most rapid increase (*P* = 0.007), but that of prisoners declined (*P* = 0.003). The prevalence of STI RG was lower than those of the other groups and remained stable throughout the ten-year period (*P* = 0.606). Percentage of anonymous tests was 2–3% of a total HIV tests, but overall HIV-positive rate showed a rapid increase (*P* < 0.001).

**Conclusions:**

As voluntary or anonymous testing groups are actively engaged in learning their status of HIV, these groups showed the highest in HIV infection. Groups in the population with these characteristics should be located and encouraged to be tested, and offered anonymity. This study suggests that it is important to ascertain the characteristics of people choosing to take voluntary testing.

## Background

Since the first individual infected with HIV was identified in South Korea in 1985, there have been a total of 8544 infected Koreans through 2011, with 7032 (82%) living with HIV. Improving survival means increased prevalence, even if incidence remains level, with a resultant public health impact and an added population disease burden. Among those Koreans infected with HIV, approximately 92% were males and most had been infected through sexual contact [1]. Most HIV tests have been conducted at public health centers (PHCs), blood centers, and hospitals. PHCs are instructed to conduct HIV screening tests for sexually transmitted infection (STI) risk group periodically and for volunteer groups arbitrarily [2]. However, the number of testing in PHCs has been reduced due to a change in the national HIV testing policy from mandatory testing to voluntary testing, since 1998 [3]. Therefore, currently only 0.4 million HIV tests (5%) are being performed in PHCs while 4.8 million HIV tests (60%) are being performed in hospitals due to both policy change and increasing number of HIV tests for surgeries, prenatal exams, health check-ups in hospitals. HIV prevalence is an important tool to describe the scale and changes in HIV infection trends in a population. Characterizing this trend is critical for evaluating policies for HIV infection prevention and management, and for predicting future public health strategy needs. Investigation of the characteristics and trends of HIV infection were done based on the number of HIV-diagnosed individuals in Korea. However, this investigation can be limited when the scope and scale in the population being tested are not considered. Therefore, a surveillance system to calculate HIV prevalence based on HIV test data has been developed and are being used since 2005. With its ready availability of record, HIV prevalence of those tested at PHCs was calculated first [2]. Next, the methods to estimate HIV prevalence of hospital patients and blood donors were developed [4,5]. Thereby, we can estimate HIV prevalence on a nationwide scale [6].

It is certain that main HIV testing institutions such as hospitals, blood centers, and PHCs encounter individuals, who are undergoing testing and share certain characteristics. The visitors to PHCs for HIV testing could have a different prevalence than those visiting hospitals or blood centers [6]. PHCs provide HIV testing not only for sexually transmitted infection (STI) risk groups, such as commercial sex workers, but also for persons wanting to be tested for HIV, local residents with low accessibility to medical care, prisoners, partners of HIV-infected individuals, and those wishing anonymous testing. PHCs are institutions that directly conduct nationally supported HIV testing and implement HIV prevention policies. Through examining the characteristics of PHCs’ visitors, an improved policy on HIV testing can be developed and immediately applied to PHCs. We use the term “visitors” throughout this paper to describe people taken HIV testing at PHCs. This study aims to investigate the population being tested for HIV at PHCs, and the annual trend and characteristics of HIV prevalence among PHCs’ visitors from 2000 onwards, when a sharp increase in the number of HIV-infected individuals was found.

## Methods

### National HIV surveillance system at PHCs

HIV screening tests at PHCs were performed by enzyme linked immunosorbent assay (ELISA, about 47%), particle agglutination (PA, about 19%), rapid method (about 25%) and others (about 9%). If a positive sample from HIV screening tests at PHCs was observed, local institutes of health & environment (IHEs) were asked to perform a confirmation of HIV infection by antigen ELISA, antibody ELISA, PA, and Western Blot. As for an indeterminate sample for HIV infection in the IHEs, HIV infection had to be confirmed by the division of AIDS of Korea Centers for Disease Control and Prevention (KCDC) [7,8]. The confirmatory test result for HIV infection was reported to the division of HIV and TB control of KCDC and was recorded in the HIV database of KCDC [8].

### HIV test data collection

PHCs manage data related to HIV testing through an electronic medical records system, namely the Health Care Information System (HCIS). The installation of HCIS program began in 2000 and was expanded to all PHCs by 2005. HIV testing data from 2000 to 2009 were collected from 253 PHCs through 16 IHEs, which are based in 7 big cities and in 9 provinces. Information collected on people being tested for HIV includes institutional code, specimen code, gender, year of birth, reason for HIV testing, HIV screening test results, differentiation code, and confirmatory HIV test code. If a HIV screening test was positive, confirmatory test code was assigned for referring confirmatory HIV testing to IHE. Confirmatory test code was composed of referring year, referring institution, and referring order. The differentiation code is a parameter to identify the frequency of tests for one individual within a year [2]. These codes avoid use of personal information and preserve patient privacy. HIV-positive data was collected from the Division of HIV and TB control of KCDC. The data include gender, year of birth, reason for HIV testing, and confirmatory test code. Data from HIV testing and HIV positive data were matched by confirmatory test code. When the data were recorded manually before the installation of HCIS program, only the number of HIV tests was obtained.

### Statistical methods

Annual HIV prevalence among visitors to PHCs was defined as the number of confirmed HIV-infected individuals per 10 thousand HIV-tested individuals at PHCs each year independently. Trends in HIV prevalence were assessed through a series of cross-sectional annual analyses. The frequency of HIV testing for one visitor was measured every year [2]. We analyzed HIV prevalence by gender, age (<20, 20–29, 30–39, 40–49, 50–59, ≥60), nationality (Korean, Foreigner), and region (Metropolis, Smaller city or rural area). A variety of reasons for HIV testing included occupation, health status, or voluntary test [1,2]. We compiled the reasons for HIV testing into 14 factors and categorized these under four groups: general group (health checkup, medical record, prenatal checkup, and others), HIV infection suspected group (HIV ISG; referral by doctor and voluntary test taker), HIV testing recommended group (HIV TRG; tuberculosis patient, prisoner, and partner of HIV-infected individual), and STI risk group (STI RG; commercial sex worker, bar employee, tea-room employee, massage parlor employee, and others) [2]. Data for anonymous group were excluded when analyzing HIV prevalence, because frequency of anonymous test for one individual within one year could not be measured. Therefore, annual HIV positive rate was calculated and defined as the number of confirmed HIV positives per 10 thousand anonymous HIV tests. Data with missing values (about 0.7%) were excluded for analyzing HIV prevalence. To assess the difference in HIV prevalence by epidemiological variables, we conducted multivariate logistic regression (gender, age, nationality, region, reason for HIV testing). Ten-year trends of HIV prevalence were analyzed using score test for trend [9,10]. All statistical analyses were performed using SAS 9.1. Ethics approval was obtained from the KCDC Institutional Review Board (IRB) Ethics Committee.

## Results

### HIV testing at PHCs

The number of HIV tests conducted at PHCs rose from around 430,000 in 2000 to 500,000 in 2004, but declined gradually from 2005 onward, averaging about 400,000 per year (Table 1). STI RG accounted for approximately 40–65% of the total number of tests taken, while the frequency of HIV testing for one individual was about 1.4 every year. The numbers of tests for HIV ISG and HIV TRG increased from 10% and 2% in 2000 to 15% and 10% in 2009, respectively. The number of anonymous tests made up about 2-3% of the total number of HIV tests every year.

**Table 1:** **HIV testing of visitors to public health centers during 2000–2009, Korea**

	**2000**	**2001**	**2002**	**2003**	**2004**	**2005**	**2006**	**2007**	**2008**	**2009**
Total no. of HIV tests	428698	487134	484028	466747	496890	353440	411017	396943	391486	395771
No. of PHCs	202	215	222	222	225	246	251	251	251	253
No. of test by HCIS (%)	129607 (30)	229006 (47)	296414 (61)	349488 (75)	435101 (88)	353440 (100)	411017 (100)	396943 (100)	391486 (100)	395771 (100)
No. of HCIS (%)	72 (36)	114 (53)	150 (68)	186 (84)	210 (93)	246 (100)	251 (100)	251 (100)	251 (100)	253 (100)
Average no. of test per PHCs	1800	2009	1796	1879	2072	1437	1638	1582	1560	1564
Frequency of test	1.11	1.15	1.25	1.30	1.31	1.23	1.23	1.22	1.22	1.20
Reason for HIV testing										
General group										
No. of test (%)	46617 (36)	80519 (35)	78452 (27)	65694 (19)	120252 (27)	90628 (26)	110394 (27)	117910 (30)	115176 (29)	123375 (31)
Frequency of test	1.02	1.03	1.03	1.04	1.04	1.03	1.04	1.04	1.04	1.04
HIV ISG										
No. of test (%)	13439 (10)	22153 (10)	33015 (11)	42786 (12)	56600 (13)	43517 (12)	56919 (14)	66544 (17)	64986 (17)	60678 (15)
Frequency of test	1.05	1.06	1.11	1.11	1.13	1.10	1.11	1.15	1.13	1.07
HIV TRG										
No. of test (%)	2288 (2)	5267 (2)	5987 (2)	8261 (2)	9058 (2)	8351 (2)	17299 (4)	22060 (6)	26864 (7)	40463(10)
Frequency of test	1.08	1.07	1.05	1.06	1.04	1.03	1.05	1.09	1.08	1.13
STI RG										
No. of test (%)	64766 (50)	116910 (51)	173096 (58)	226155 (65)	242369 (56)	201067 (57)	215459 (52)	180820 (45)	175864 (45)	162278 (41)
Frequency of test	1.18	1.27	1.39	1.43	1.44	1.39	1.39	1.40	1.43	1.43
Anonymous										
No. of test (%)	2497 (2)	4157 (2)	5864 (2)	6592 (2)	6822 (2)	9877 (3)	10946 (3)	9609 (2)	8595 (2)	8977 (2)

Total no. of HIV test was collected by HCIS (health care information system) including documentation from PHCs.

PHCs; Public health centers, No. of HCIS: The number of PHCs with HCIS.

General group: HIV test takers for health checkup, medical certification, and prenatal check up.

HIV ISG (HIV infection suspected group) included HIV test takers additional HIV test through referral by doctor and voluntary test taker.

HIV TRG (HIV test recommended group) was composed of TB patient, prisoner, and partner of HIV-infected individual.

STI RG (sexually transmitted infection risk group) was composed between commercial sex workers, bar employees, tea-room employees, and massage parlor employees.

Frequency of test: The number of HIV-testing per PHC visitor per year.

Frequency of test of anonymous testers could not be calculated, because we were unable to calculate the number of tests per individuals.

### Trend in HIV prevalence among PHCs’ visitors

The annual HIV prevalence among PHCs’ visitors by gender, age, nationality, region, and reason for HIV testing are presented in Table 2. Gender and age distribution among PHCs’ visitors is shown to be large in women (averaging 70%) and in the 20s (approximately 40%). HIV prevalence went up from 1.3 per 10 thousand individuals in 2000 to 5.3 per 10 thousand individuals in 2005 (*P* < 0.001); however, the prevalence decreased to under 4.5 per 10 thousand individuals after 2005 and was stable thereafter (*P* = 0.200). The number of HIV tests among foreigners was small, but their prevalence was significantly higher than that of Koreans (*P* < 0.001 in 2000, *P* = 0.053 in 2009). Men had higher prevalence than women (*P* < 0.001), and the 40s had higher prevalence than other age groups (*P* < 0.001). The prevalence of men (*P* = 0.017), test takers in the 20s (*P* = 0.001) and those aged over 40 years (40s; *P* = 0.038, 50s; *P* = 0.041, 60s; *P* = 0.042) increased significantly over the years. Although the 30s tended to show the highest prevalence until 2003, they presented lower prevalence than the 40s starting from 2004 onwards, and currently showed lower prevalence than the 50s.

**Table 2:** **Characteristics of trends in HIV prevalence of test takers in public health centers during 2000–2009 in Korea**

**Category**	**2000**		**2001**		**2002**		**2003**		**2004**	
	**N (HIV+)**	**Prevalence (95%CI)**	**N (HIV+)**	**Prevalence (95%CI)**	**N (HIV+)**	**Prevalence (95%CI)**	**N (HIV+)**	**Prevalence (95%CI)**	**N (HIV+)**	**Prevalence (95%CI)**
Total	109373 (14)	1.3 (0.6-2.0)	187264 (35)	1.9 (1.2-2.5)	223183 (69)	3.1 (2.4-3.8)	255849 (82)	3.2 (2.5-3.9)	332405 (146)	4.4 (3.7-5.1)
Gender										
Women	74817 (3)	0.4 (0.0-0.9)	129380 (12)	0.9 (0.4-1.5)	156380 (11)	0.7 (0.3-1.1)	186954 (14)	0.7 (0.4-1.1)	226611 (22)	1.0 (0.6-1.4)
Men	34556 (11)	3.2 (1.3-5.1)	57884 (23)	4.0 (2.3-5.6)	66803 (58)	8.7 (6.4-10.9)	68895 (68)	9.9 (7.5-12.2)	105794 (124)	11.7 (9.7-13.8)
Age										
<20	8963 (0)	0.0	13688 (0)	0.0	18047 (2)	1.1 (0.0-2.6)	17805 (1)	0.6 ( 0–1.7)	16817 (3)	1.8 (0.0-3.8)
20-29	50442 (4)	0.8 (0.0-1.6)	88100 (10)	1.1 (0.4-1.8)	110668 (19)	1.7 (0.9-2.5)	132136 (28)	2.1 (1.3-2.9)	145146 (36)	2.5 (1.7-3.3)
30-39	24718 (9)	3.6 (1.3-6.0)	41323 (13)	3.1 (1.4-4.9)	48334 (31)	6.4 (4.2-8.7)	57432 (36)	6.3 (4.2-8.3)	85181 (56)	6.6 (4.9-8.3)
40-49	13523 (1)	0.7 (0.0-2.2)	23519 (10)	4.3 (1.6-6.9)	26043 (10)	3.8 (1.5-6.2)	29509 (10)	3.4 (1.3-5.5)	48873 (36)	7.4 (5.0-9.8)
50-59	6256 (0)	0.0	11070 (2)	1.8 (0.0-4.3)	10255 (7)	6.8 (1.8-11.9)	10023 (1)	1.0 (0.0-3.0)	22279 (10)	4.5 (1.7-7.3)
60≤	5471 (0)	0.0	9564 (0)	0.0	9836 (0)	0.0	8944 (6)	6.7 (1.3-12.1)	14109 (5)	3.5 (0.4-6.6)
Nationality										
Korean	108957 (7)	0.6 (0.2-1.1)	186666 (28)	1.5 (0.9-2.1)	221508 (58)	2.6 (1.9-3.3)	253183 (75)	3.0 (2.3-3.6)	278582 (98)	3.5 (2.8-4.2)
Foreign	416 (7)	168 (45–292)	598 (7)	117 (31–203)	1675 (11)	65.7 (27–105)	2666 (7)	26.3 (6.8-45.7)	53823 (48)	8.9 (6.4-11.4)
Region										
Metropolis	41357 (6)	1.5 (0.3-2.6)	78960 (17)	2.2 (1.1-3.2)	84147 (41)	4.9 (3.4-6.4)	114531 (46)	4.0 (2.9-5.2)	139024 (81)	5.8 (4.6-7.1)
Smaller city or rural area	68016 (8)	1.2 (0.4-2.0)	108304 (18)	1.7 (0.9-2.4)	139036 (28)	2.0 (1.3-2.8)	141318 (36)	2.5 (1.7-3.4)	193381 (65)	3.4 (2.5-4.2)
Reason for HIV testing										
General group	43752 (8)	1.8 (0.6-3.1)	73190 (13)	1.8 (0.8-2.7)	66609 (17)	2.6 (1.3-3.8)	55003 (22)	4.0 (2.3-5.7)	109440 (45)	4.1 (2.9-5.3)
HIV ISG	12357 (4)	3.2 (0.1-6.4)	20255 (9)	4.4 (1.5-7.3)	28806 (38)	13.2 (9.0-17.4)	37325 (46)	12.3 (8.8-15.9)	49145 (78)	15.9 (12.4-19.4)
HIV TRG	2127 (2)	9.4 (0.0-22.4)	4776 (8)	16.8 (5.2-28.3)	5512 (8)	14.5 (4.5-24.6)	7487 (7)	9.3 (2.4-16.3)	8664 (8)	9.2 (2.8-15.6)
STI RG	51137 (0)	0.0	89043 (5)	0.6 (0.1-1.1)	122256 (6)	0.5 (0.1-0.9)	156034 (7)	0.4 (0.1-0.8)	165156 (15)	0.9 (0.4-1.4)
**2005**		**2006**		**2007**		**2008**		**2009**		P-value*
**N (HIV+)**	**Prevalence (95%CI)**	**N (HIV+)**	**Prevalence (95%CI)**	**N (HIV+)**	**Prevalence (95%CI)**	**N (HIV+)**	**Prevalence (95%CI)**	**N (HIV+)**	**Prevalence (95%CI)**	
280456(149)	5.3 (4.5-6.5)	317451(132)	4.2 (3.4-4.9)	306898 (126)	4.1 (3.4-4.8)	307588 (132)	4.3 (3.6-5.0)	321356 (141)	4.4 (3.7-5.1)	0.012
192687(25)	1.3 (0.8-1.8)	220172 (21)	1.0 (0.5-1.4)	208425 (16)	0.8 (0.4-1.1)	204156 (18)	0.9 (0.5-1.3)	211064 (22)	1.0 (0.6-1.5)	0.106
87769(124)	14.1(11.6,16.6)	97279 (111)	11.4 (9.3-13.5)	98473 (110)	11.2 (9.1-13.3)	103432 (114)	11.0 (9.0-13.0)	110292 (119)	10.8 (8.8-12.7)	0.017
15587(4)	2.6 (0.1-5.1)	16165 (2)	1.2 (0–3.0)	16883 (1)	0.6 (0–1.8)	18221 (2)	1.1 (0–2.6)	19982 (4)	2.0 (0.0-4.0)	0.094
127393(35)	2.7 (1.8-3.7)	132362 (34)	2.6 (1.7-3.4)	120786 (24)	2.0 (1.2-2.8)	114155 (36)	3.2 (2.1-4.2)	109797 (41)	3.7 (2.6-4.9)	0.001
70723(56)	7.9 (5.8-10.0)	89113 (43)	4.8 (3.4-6.3)	88610 (46)	5.2 (3.7-6.7)	86969 (46)	5.3 (3.8-6.8)	94523 (41)	4.3 (3.0-5.7)	0.612
37559(41)	10.9 (7.6-14.3)	48626 (39)	8.0 (5.5-10.5)	45210 (35)	7.7 (5.2-10.3)	49715 (31)	6.2 (4.0-8.4)	51717 (31)	6.0 (3.9-8.1)	0.038
15196(8)	5.3 (1.6-8.9)	17652 (8)	4.5 (1.4-7.7)	19365 (12)	6.2 (2.7-9.7)	23351 (12)	5.1 (2.2-8.0)	26368 (15)	5.7 (2.8-8.6)	0.041
13998(5)	3.6 (0.4-6.7)	13533 (6)	4.4 (0.9-8.0)	16044 (8)	5.0 (1.5-8.4)	15177 (5)	3.3 (0.4-6.2)	18969 (6)	4.7 (1.6-7.8)	0.042
270497(131)	4.8 (4.0-5.7)	304348 (120)	3.9 (3.2-4.6)	291015 (111)	3.8 (3.1-4.5)	287921 (114)	4.0 (3.2-4.7)	299466 (126)	4.2 (3.5-4.9)	0.002
9959(18)	18.1 (9.7-26.4)	13103 (12)	9.2 (4.0-14.3)	15883 (15)	9.4 (4.7-14.2)	19667 (18)	9.2 (4.9-13.4)	21890 (15)	6.8 (3.4-10.3)	0.022
113290(87)	7.7 (6.1-9.3)	119995 (87)	7.3 (5.7-8.8)	112885 (67)	5.9 (4.5-7.4)	113793 (84)	7.4 (5.8-9.0)	120597 (92)	7.6 (6.1-9.2)	0.001
167166(62)	3.7 (2.8-4.6)	197456 (45)	2.3 (1.6-2.9)	194013 (59)	3.0 (2.3-3.8)	193795 (48)	2.5 (1.8-3.2)	200759 (49)	2.4 (1.8-3.1)	0.094
88116 (32)	3.6 (2.4-4.9)	101633 (24)	2.4 (1.4-3.3)	109670 (30)	2.7 (1.8-3.7)	108003 (23)	2.1 (1.3-3.0)	114589 (24)	2.1 (1.3-2.9)	0.921
39502(86)	21.8 (17.2-26.4)	48567 (87)	17.9 (14.2-21.7)	54755 (81)	14.8 (11.6-18.0)	54108 (93)	17.2 (13.7-20.7)	55360 (91)	16.4 (13.1-19.8)	0.019
8144(10)	12.3 (4.7-19.9)	16161 (13)	8.0 (3.7-12.4)	17019 (10)	5.9 (2.2-0.7)	25098 (9)	3.6 (1.2-5.9)	37319 (21)	5.6 (3.2-8.0)	0.010
144694(21)	1.5 (0.8-2.1)	151090 (8)	0.5 (0.2-0.9)	125454 (5)	0.4 (0.0-0.7)	120379 (7)	0.6 (0.2-1.0)	114088 (5)	0.4 (0.1-0.8)	0.606

N: the number of HIV-tested individuals, HIV+: the number of HIV-infected individuals.

Total (The total number of HIV-tested individuals) was calculated without data of anonymous test.

Prevalence (HIV prevalence): the number of HIV-infected individuals per 10 thousand HIV-tested individuals, CI: confidence interval.

General group: HIV test takers for health checkup, medical certification and prenatal check up.

HIV ISG (HIV infection suspected group) included HIV test takers additional HIV test through referral by doctor and voluntary test taker.

HIV TRG (HIV test recommended group) was composed of TB patient, prisoner, and partner of HIV-infected individuals.

STI RG (sexually transmitted infection risk group) was composed of commercial sex worker, bar employee, tea-room employee, massage parlor employee.

* P-value by adjusted score test for trend of HIV prevalence for 10 years.

Testing at PHCs in metropolis accounted for approximately 40% of a total tests and the prevalence was higher than those in smaller cities or rural areas.

### Trend in HIV prevalence among PHCs’ visitors by reason for HIV testing

**Figure 1:**
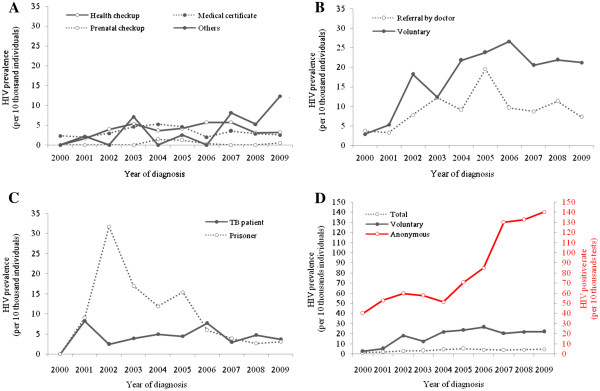
**The trend in HIV prevalence or positive rate by reason for HIV testing.** (**A**) General Group: Health checkup (*P* = 0.134), Medical record (*P* = 0.776), Prenatal checkup (*P* = 0.623), Others (*P* = 0.039) (**B**) HIV ISG (HIV infection suspected group): Referral by doctor (P = 0.226), Voluntary (voluntary test taker, *P* = 0.007), (**C**) HIV TRG (HIV test recommended group): TB patients (*P* = 0.069), Prisoners (*P* = 0.003, excluded 2000 and 2001), (**D**) Total (a total HIV test takers, *P* = 0.012), Voluntary (voluntary test takers, *P* = 0.007), and Anonymous (anonymous test takers, *P* < 0.001). HIV TRG included partners of HIV-infected individuals, but it was not presented in figure C, because their prevalence was much higher compare to those of other groups (the number of HIV-infected individuals/the number of HIV-tested individuals, 2000: 2/3, 2001:4/6, 2002: 2/5, 2003: 1/6, 2004: 2/8, 2005: 5/27, 2006: 2/18, 2007: 4/24, 2008: 1/39, 2009: 9/36). In figure D, anonymous test takers present HIV positive rate instead of HIV prevalence. *P-value by score test for trend of HIV prevalence for ten years.

The annual trend of HIV prevalence by reason for HIV testing among PHCs’ visitors is shown in Table 2 and Figure 1. From 2000 to 2004, the prevalence was increased from 1.8 per 10 thousand individuals to 4.1 per 10 thousand individuals (*P* = 0.021) among general group (Table 2). Since 2004 the prevalence has decreased to 2.1 per 10 thousand individuals (*P* = 0.023). The prevalence in HIV ISG rose from 3.2 per 10 thousand individuals in 2000 to 21.8 per 10 thousand individuals in 2005 (*P* = 0.002) and has been stable at approximately 17.0 per 10 thousand individuals since 2006. Although the prevalence in HIV TRG was high during the first three years after 2000, it has reduced overall since then (*P* = 0.010). The prevalence in STI RG was analysed as lower than among other groups, and was not statistically significantly different by year (*P* = 0.606).

When the prevalence was examined in detail by reason for HIV testing (Figure 1). The prevalence among voluntary test takers with concerns about HIV infection rose dramatically from 2.9 per 10 thousand individuals in 2000, to 21.2 per 10 thousand individuals in 2009 (*P* = 0.007); this group showed the highest prevalence except for partners of infected individuals. A total of 172 partners of HIV-infected individuals were tested during the study period and 32 (18.6%) were diagnosed with HIV infection (data not shown). For HIV TRG, the prevalence among prisoners decreased from 31.7 per 10 thousand individuals in 2002 to 3.1 per 10 thousand individuals in 2009 (*P* = 0.003), and there was a decrease in HIV prevalence among tuberculosis patients treated at PHCs (*P* = 0.069). The positive rate of anonymous test takers increased sharply from 40.0 per 10 thousand tests in 2000, to 140.4 per 10 thousand tests in 2009 (*P* < 0.001) and was significantly higher than the prevalence of voluntary (*P* < 0.001). In general group, there was no increase in prevalence among the subgroups such as health checkup (*P* = 0.134), medical record (*P* = 0.776) and prenatal checkup (*P* = 0.623). Employees at bars and tea rooms were composed of over 80% of the STI RG, and HIV-infected individuals were diagnosed primarily among bar employees (data not shown).

## Discussion

This cross-sectional study analyzed the trend and characteristics in HIV prevalence of PHCs’ visitors by successive annual surveillance. Our findings revealed that HIV prevalence increased rapidly during the first six years from 2000 and has been stable after first decline. For group by reasons for HIV testing, the prevalence of voluntary test takers was higher than those of the other groups. Their prevalence increased to 30 per 10 thousand individuals in 2006 and remained stable at about 21 per 10 thousand individuals since then. Another main finding shows very high HIV positive rate of anonymous tests with increasing trend during the ten-year period. Beside positives in the anonymous, the number of HIV-diagnosed individuals in South Korea rose every year from 244 in 2000 to 839 in 2009, and the ratio of HIV-diagnosed men to women rose from 6:1 in 2000 to 9:1 in 2009. For the age at diagnosis, the 30s showed the highest proportion of HIV-infection overall, but an increase was seen in older age groups [1]. The patterns of men to women ratio, distribution of age at diagnosis, and annual change in the number of HIV-diagnosed individuals in South Korea were similarly observed with those of the prevalence of PHCs’ visitors. The number of HIV screening tests conducted at PHCs comprised of only about 5% of the total HIV tests performed in the country [4,5], but 18% of all infected individuals who were newly diagnosed with HIV were found by screening test at PHCs. A previous study on reasons for HIV testing among HIV-diagnosed individuals in South Korea has found that the large number of positives at PHCs took the tests voluntarily [11].

Among persons who voluntarily tested for HIV, men were more than women and men in the 30s and the 40s were more than those in other age groups. Some of the voluntary PHCs’ visitors took the tests anonymously. Their prevalence was not known, but their HIV positive rate increased to 140 per 10 thousand HIV tests in 2009. The recent increase in the positive rate was partly due to the promotion of anonymous HIV tests, as a tool to encourage HIV testing in Korea. For promotion of taking the HIV test anonymously, institutions performing anonymous HIV testing were expanded to hospitals in 2008. When anonymous test takers are diagnosed as HIV positive, they are informed of the national support for HIV treatments and are asked to choose whether to register with the national support program [8]. Around 25% of the total positive cases by the anonymous tests registered with this program [1]. These findings suggest that the quality of HIV testing service should be enhanced to ensure that persons with a high risk of HIV infection undergo the tests more actively to prevent the spread of HIV infection. Continuous education and public relations should also be enhanced to improve accessibility through a long-term effort to change the perception of persons who avoid testing due to their fear of identity disclosure.

The proportion of foreigners being tested was so low, but foreigners showed much higher HIV prevalence than Koreans. The scale of HIV testing for foreigners has increased because they underwent required testing at PHCs in the process of obtaining alien registration [12]. Of the foreigners tested at PHCs, 60% were Asians and 20% were Africans, and approximately 60% of them underwent testing to obtain medical records for work (data not shown). Foreigners undergo HIV testing for several reasons. First, when foreigners reside in the country for over three months for the purpose of entertainment and sports, they have to submit recent HIV test certificates (HIV negative result tested within one month) upon entering the country or undergo HIV testing within 72 hours if they do not submit a test certificate [13]. Second, foreign workers entering the country through the employment permit system must undergo HIV testing during the training period for works [14]. The number of foreigners in Korea has increased to approximately 1.3 million in 2010 [15]. Social, economic and political factors of their countries and Korea influence the risk of HIV infection of foreign workers, which is exacerbated by limited access to HIV testing services and fear of being stigmatized when seeking HIV-related information or support [16]. Therefore, public relations and education concerning HIV prevention for foreigners have been promoted and offered through various mass media and communities by various countries in Korea. Foreigners can undergo HIV testing anonymously at PHCs and hospitals and they can receive counseling at two HIV counseling centers for foreigners before and after the tests.

STI RG, who test for HIV regularly, was approximately 50% of PHCs’ visitors; 90% of them were women and 65% of them were less than 30 years old. The HIV prevalence of STI RG was lower than those among other groups and the prevalence of women in STI RG was significantly low (about 0.3 per 10 thousand individuals). Men was about 10% of STI RG and their HIV prevalence was about 3.9 per 10 thousand individuals (data not shown). As HIV testing policy related to STI RG has changed, the scale of HIV tests at PHCs has changed as well. HIV testing for STI RG has been performed regularly every six months since 1986 in Korea. Regularly tested groups (CSWs, bar and tearoom employees, and massage parlor employees) were kept and expanded to food industry employees, restaurant employees, and others, so the number of tests at PHCs has increased every year [17]. After a periodic examination program (registration program) of STD was abolished in 1998, regular HIV testing for food industry employees, restaurants employees became a self-initiated test, so the scale of testing at PHCs dropped [3]. Special laws against prostitution enforced starting in October 2004 [18] resulted in a small decrease in the number of HIV tests for STI RG at PHCs since 2005, but there was no difference in prevalence by year. This finding can be useful to evaluate the outcomes of the national testing policy for the STI RG. For HIV TRG, the very high prevalence of partners of HIV-infected individuals showed the importance of regular HIV testing of these persons in Korea. Though the prevalence among prisoners was sharply decreasing, it is still necessary to monitor them. HIV prevalence of tuberculosis patients was similar to those of health checkup and medical records among general group (respectively *P* = 0.775, *P* = 0.144 in 2009).

This study has several limitations. As HCIS program was installed at all PHCs in 2005, the prevalence before 2005 was estimated and its calculation was only based on data of 72–210 PHCs with HCIS program. Therefore, the prevalence before 2005 represents a smaller population with biases rather than the whole population since 2005. In addition, HIV positive rate of tests taken by an individual anonymously; was not considered when obtaining HIV prevalence.

## Conclusions

This study provides useful data to identify characteristics and trends in HIV prevalence among PHCs’ visitors for the ten most recent years in South Korea. These results are useful to scientifically evaluate the recent national HIV prevention policies. The HIV prevalence for specific groups, such as regular HIV testing groups or foreigners, can be used to estimate the nationwide scale of HIV-infected individuals and to predict the future trends of HIV infection [19]. Voluntary testing, including anonymous testing, was shown to be more effective in detecting HIV infection. This study showed that it is important to develop the anonymous testing strategy to encourage people to take HIV test voluntarily. Those submitting to voluntary testing, including anonymous testing, showed the highest HIV infection. This study suggests that it is important to ascertain the characteristics of people choosing to have voluntary testing. Groups in the population with these characteristics should be located and encouraged to be tested, and offered anonymity. In addition, the data can be a useful source not only for creating future projects for HIV prevention, but also for establishing policies to prevent the spread of HIV in South Korea.

## Competing interests

The authors declare that they have no competing interests.

## Authors’ contributions

Mee-Kyung Kee designed and conceived the idea for the study and wrote the first draft of the manuscript and coordinated funding for the project HIV/AIDS seroprevalence in Korea. Jin-Hee Lee designed and conceived the idea for the study and completed all data analyses and interpretation of data. Jiyoung Hwang contributed to collection of epidemiological data of HIV-diagnosed individuals. Sung Soon Kim supervised all aspects of its implementation coordinated funding for the project and contributed to the critical revising for important intellectual content. All authors read and approved the final version of the manuscript as submitted to BMC Public Health.

## Pre-publication history

The pre-publication history for this paper can be accessed here:

http://www.biomedcentral.com/1471-2458/12/831/prepub
